# A Case of Artificial Snow Foam induced Corneal Endotheliitis Followed up by Scheimpflug Densitometry

**Published:** 2019

**Authors:** Amr MOUNIR, Omar Fawzy ZIDAN, Islam AWNY, Engy Mohamed MOSTAFA

**Affiliations:** 1 Department of Ophthalmology, Sohag Faculty of Medicine, Sohag University, Sohag, Egypt; 2 Sohag Ophthalmology Hospital, Sohag, Egypt

**Keywords:** Cornea, Scheimpflug Densitometry, Snow Foam, Polymerase Chain Reaction, Corneal Endotheliitis

## Abstract

The aim was to present a rare case of artificial snow foam induced corneal endotheliitis followed up by Scheimpflug Densitometry. A 15-year-old male complained of redness, tearing and reduced vision in the left eye after artificial snow foam entered his left eye 4 days before the presentation. Slit lamp examination of the same eye showed ciliary injection with corneal edema with no epithelial defect and endothelial lesion measuring 3 × 4 millimeters (mm) with large keratic precipitates (KP). Examining the left eye by the Scheimpflug densitometry of the Sirius device (CSO, Florence, Italy) showed plaque on the back of the cornea. Aqueous tab Polymerase chain reaction analysis (PCR) results for the affected eye had negative results for viral infection. Improvement of ocular symptoms occurred after treatment with topical steroid therapy. Scheimpflug densitometry showed disappearance of the saw-tooth protrusions on the back of the cornea with decreased reflectivity. Corneal endotheliitis can be triggered by chemical ocular trauma. The Scheimpflug densitometry examination may be a useful noninvasive method for reaching a clinical diagnosis of corneal endotheliitis and monitoring treatment effectiveness.

## INTRODUCTION

Acute corneal edema could be due to a myriad of conditions such as angle closure glaucoma, post-intraocular surgery and trauma [1]. Chemical trauma is very common especially in children with increased risk of vision compromise [2]. Chemical injury derives its bad reputation from its speedy effect and high penetrance to intraocular tissues resulting in irreversible damage [3]. While in patients without former history of surgery, trauma or noxious agents, it is known to be initiated by infectious agents especially viruses such as Cytomegalovirus (CMV) [4], Varicella zoster [5] and Herpes simplex virus (HSV) [6]. Scheimpflug densitometry which is performed by corneal tomography by a rotating Scheimpflug camera is a simple and effective diagnostic procedure for the anterior segment of the eye especially the corneal media. This maneuver allows quantitative evaluation of the optical media as “densitometry” [7]. Many clinical studies revealed the importance of densitometry as a new diagnostic method for evaluation of corneal opacification density in various clinical conditions [8-10]. The aim of this report was to present a rare case of artificial snow foam induced corneal endotheliitis diagnosed by Scheimpflug Densitometry.

## CASE REPORT

A 15-year-old male presented to our outpatient clinic complaining of pain, redness, tearing and reduced vision in the left eye after artificial snow foam entered his left eye 4 days before (June 2018). The boy had a contact with this substance in a wedding ceremony. 

**Figure 1:**
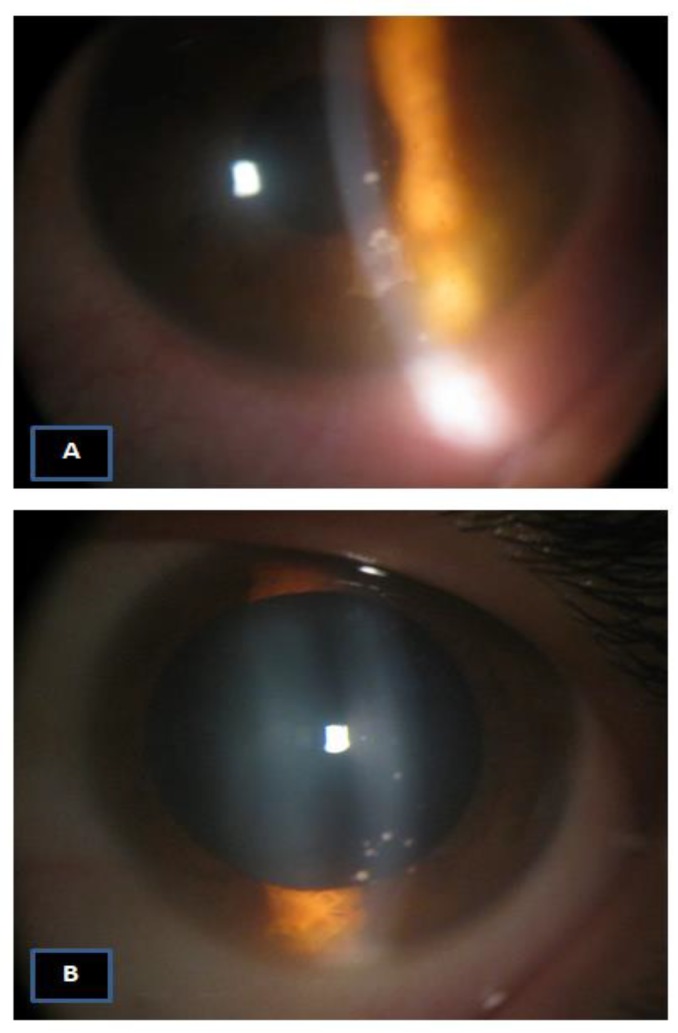
A) Stellate shape endothelial lesion measuring 3 × 4 mm with large keratic precipitates (KPs). B) Endothelial plaque disappeared, but fine KPs still remained.

**Figure 2:**
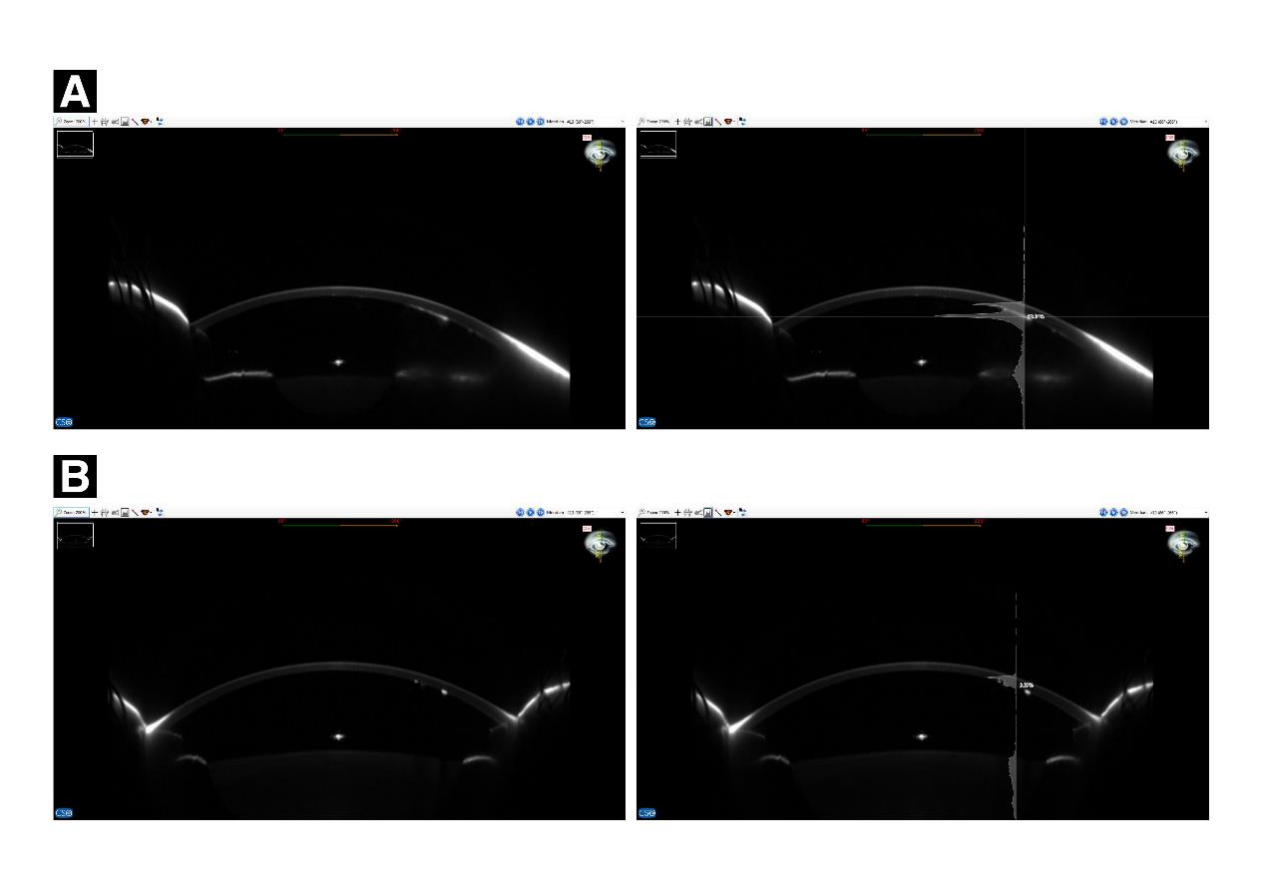
A) Scheimpflug densitometry showing plaque on the back of the cornea with high reflectivity. B) Scheimpflug densitometry showing disappearance of the saw-tooth protrusions on the back of the cornea with decreased reflectivity. However, fine hyper-reflective dots still remained.

Written informed consent was obtained from the patient to publish this case report. History taking did not reveal any medical history either systemic or ocular. His parents reported that at the time of exposure, they washed his eyes profusely with tap water for unspecified amount of time. He did not seek medical advice as he did not complain except for redness which was attributed to the snow spray. No PH measurement was available as the patient presented 4 days after presentation. On examination, the corrected distance visual acuity (CDVA) in the right eye was 6/6 and in the affected eye was 6/24. Examination of the right eye by Slit lamp was unremarkable while the examination of the left eye showed ciliary injection with corneal edema with no epithelial defects. Yet there was a stellate shape endothelial lesion measuring 3 × 4 millimeters (mm) with large keratic precipitates (KP). The KPs were rounded, sharply marginated and pigmented deposited at the center and inferior part of the cornea (Fig. 1 A). The anterior chamber showed mild cell and flare. Examination of the posterior segment of both eyes were within the normal limits. Intraocular pressure (IOP) was normal. Examining the left eye by the Scheimpflug densitometry of the Sirius device (CSO, Florence, Italy), a plaque on the back of the cornea was evident. There was a protruding mass at the posterior corneal surface with a saw-tooth appearance and the density of the structure images was high. The reflectivity of the posterior corneal surface including the endothelium was high (Fig. 2A). Corneal thickness was 691 micrometers.

The patient was investigated for underlying immunosuppressive causes and was found to have normal routine investigations and negative results for human immunodeficiency virus (HIV) and the Venereal Disease Research Laboratory test (VDRL). An anterior chamber paracentesis was performed for aqueous tap for the left eye and was sent for Polymerase chain reaction analysis (PCR) of Varicella zoster virus, HSV, and CMV, which had negative results for all the three viruses. Our approach was to start with prednisolone acetate 1% (Predforte. Alcon) every two hours with cyclopentolate eyedrops (cyclopherin) 3 times daily, moxifloxacillin hydrochloride ophthalmic solution 0.5% drops 4 times a day and tobramycin- prednisolone eye ointment at nights.

Within 3 days, the patient presented with rapid relieve of signs with a quiet eye. The endothelial plaque and KPs decreased in size and number. The corneal edema decreased as well (Fig. 1B). Visual acuity improved to 6/12. A week later, the eye was free of inflammation with only a few KPs present. Follow up of densitometry scans showed decrease of hyper-reflectivity of the posterior surface of the cornea and disappearance of the saw-tooth protrusions (Fig. 2 B) and the corneal thickness was below 530 micrometers. The treatment was tapered over a month with no report of recurrence. An informed consent was acquired from the patient's parents and the ethical committee of the Sohag refractive center was acquired to report the case.

## DISCUSSION

Ocular chemical injury is common and can cause variable ocular damage based on the nature and type of chemical substance [3]. Fake snow that was the reason of injury in this patient is composed of sodium polyacrylate granules which are converted to snow-like semi-sticky matter when exposed to water causing damage to the corneal epithelium due to its acidic nature [11]. Our patient did not experience any epithelial defect or erosions which made the diagnosis of chemical keratouveitis not the first on our list. Negative aqueous tap excluded viral endotheliitis as well. Also, this led us to consider other causes of unilateral acute corneal edema without epithelial compromise such as affection of endothelial dysfunction or elevation in IOP [12]. Posterior corneal dystrophies or iridocorneal syndrome were not in the differential diagnosis due to their chronicity [13, 14]. However, we could not exclude the liability that this chemical accident might trigger any pathology. Aqueous tab result had negative result which guided us that the cause of corneal endotheliitis was autoimmune in nature or due to chemical keratouveitis.

In the United States, herpetic disciform keratitis is considered as endotheliitis due to the presence of KPs and edema [15, 16], while in Japan, herpetic disciform keratitis is considered as stromal keratitis which is a combination of stromal keratitis and endotheliitis [17]. Cytomegalovirus corneal endotheliitis is different from CMV retinitis in that it occurs in immunocompetent healthy patients. Cytomegalovirus corneal endotheliitis can be diagnosed by detection of CMV DNA using aqueous humor PCR along with positive response to anti-CMV drugs, such as ganciclovir and valganciclovir [17]. We did not use systemic antiviral therapy or topical ganciclovir due to the negative results of anterior chamber paracentesis which supported active nature of the keratouveitis. The mechanism of corneal endotheliitis is considered to be autoimmune due to its similarity with graft rejection and its good response to topical corticosteroid therapy. Abulafia et al. [18] reported 135 eyes with similar party foam contact, yet none developed similar condition. They only reported chemical conjunctivitis in 100% of eyes and superficial punctate keratopathy in 79% of eyes, corneal erosion in 27% of eyes and finally conjunctival erosion in 5% [18]. The Scheimpflug densitometry was used for diagnosis and follow-up of our case where changes in reflectivity of the posterior surface of the cornea were the guide for us in monitoring the progress of endotheliitis. The Scheimpflug images acquired were similar to images taken by anterior segment Optical coherence tomography (OCT) in studies by Kobayashi et al [19]. in cases of CMV endotheliitis. Many studies [20-23] used the Scheimpflug densitometry as a tool for corneal optical density but our case was the first one to use this relatively available procedure to evaluate corneal endothelial lesions.

## CONCLUSIONS

Corneal endotheliitis can be triggered by chemical ocular trauma. The Scheimpflug densitometry examination may be a useful noninvasive method for reaching a clinical diagnosis of corneal endotheliitis and monitoring treatment effectiveness.
